# Integrative analysis of a necroptosis-related gene signature of clinical value and heterogeneity in diffuse large B cell lymphoma

**DOI:** 10.3389/fgene.2022.911443

**Published:** 2022-08-11

**Authors:** Yu-Biao Pan, Wei Wang, Hong-Kai Cai, Jia Zhang, Ya Teng, Jiji Xue, Min Zhu, Wen-Da Luo

**Affiliations:** ^1^ Taizhou Hospital of Zhejiang Province, Zhejiang University, Hangzhoua, China; ^2^ Department of Hematology, Taizhou Hospital of Zhejiang Province Affiliated to Wenzhou Medical University, Linhai, China; ^3^ Taizhou Hospital of Zhejiang Province Affiliated to Wenzhou Medical University, Linhai, China

**Keywords:** diffuse large B-cell lymphoma, necroptosis, prognosis, TME, immunization

## Abstract

**Background:** Diffuse large B-cell lymphoma (DLBCL), which is considered to be the most common subtype of lymphoma, is an aggressive tumor. Necroptosis, a novel type of programmed cell death, plays a bidirectional role in tumors and participates in the tumor microenvironment to influence tumor development. Targeting necroptosis is an intriguing direction, whereas its role in DLBCL needs to be further discussed.

**Methods:** We obtained 17 DLBCL-associated necroptosis-related genes by univariate cox regression screening. We clustered in GSE31312 depending on their expressions of these 17 genes and analyzed the differences in clinical characteristics between different clusters. To investigate the differences in prognosis across distinct clusters, the Kaplan-Meier method was utilized. The variations in the tumor immune microenvironment (TME) between distinct necroptosis-related clusters were investigated via “ESTIMATE”, “Cibersort” and single-sample geneset enrichment analysis (ssGSEA). Finally, we constructed a 6-gene prognostic model by lasso-cox regression and subsequently integrated clinical features to construct a prognostic nomogram.

**Results:** Our analysis indicated stable but distinct mechanism of action of necroptosis in DLBCL. Based on necroptosis-related genes and cluster-associated genes, we identified three groups of patients with significant differences in prognosis, TME, and chemotherapy drug sensitivity. Analysis of immune infiltration in the TME showed that cluster 1, which displayed the best prognosis, was significantly infiltrated by natural killer T cells, dendritic cells, CD8^+^ T cells, and M1 macrophages. Cluster 3 presented M2 macrophage infiltration and the worst prognosis. Importantly, the prognostic model successfully differentiated high-risk from low-risk patients, and could forecast the survival of DLBCL patients. And the constructed nomogram demonstrated a remarkable capacity to forecast the survival time of DLBCL patients after incorporating predictive clinical characteristics.

**Conclusion:** The different patterns of necroptosis explain its role in regulating the immune microenvironment of DLBCL and the response to R-CHOP treatment. Systematic assessment of necroptosis patterns in patients with DLBCL will help us understand the characteristics of tumor microenvironment cell infiltration and aid in the development of tailored therapy regimens.

## Introduction

DLBCL is an aggressive form of lymphoma that accounts for roughly 33 percent of all non-Hodgkin lymphomas (NHL) diagnosed each year (Liu and Barta, 2019; Siegel et al., 2019). Approximately two-thirds of patients with DLBCL are alleviated by the standard R-CHOP regimen that includes rituximab, cyclophosphamide, adriamycin, vincristine, and prednisolone. However, approximately one-third of patients present unsatisfactory results with this treatment; hence, long-term remission is achieved only in a small number of patients, implying that novel molecular targets for DLBCL therapy are urgently needed, implying that novel molecular indicators for the therapy of DLBCL patients are desperately needed (Coiffier et al., 2010; Sehn and Gascoyne, 2015).

Necroptosis is a new kind of programmed cell death that combines apoptosis with necrosis. It is primarily mediated by receptor-interacting protein kinase 1 (RIPK1), receptor-interacting protein kinase 3 (RIPK3), and mixed lineage kinase domain-like pseudokinase (MLKL) (Degterev et al., 2008; Pasparakis and Vandenabeele, 2015). Currently, necroptosis is found to be closely associated with tumors, and this relationship is two-sided. On the one hand, it can act as a programmed cell death to inhibit tumorigenesis and development; on the other hand, necroptosis can activate pro-inflammatory signals that strengthen cancer cells’ proliferation and metastasis and enhance their invasiveness (McCormick et al., 2016; Strilic et al., 2016). One study found that in RIPK3 knockout mice, unregulated activation of some signaling pathways, such as the NF-kB and Wnt-β-catenin protein pathways, enhanced the capacity of intestinal epithelial cells (IEC) to multiply abnormally in a continuous inflammatory microenvironment, hence accelerating colorectal carcinogenesis (Bozec et al., 2016); Feng X et al. discovered that patients of colorectal cancer with low RIP3 expression had a lower overall survival (OS) and progression-free survival (PFS) than those that expressed high level of RIP3, insinuating that RIPK3 as a predictor of survival (Feng et al., 2015). Subsequent *in vitro* experiments demonstrated that overexpression of RIP3 significantly stunted the proliferation of cancer cells; similar to the above reports, Ertao et al. (2016), Sun et al. (2019) found that the down-regulation of MLKL was significantly associated with reduced OS in gastric cancer (GC) patients, implying that MLKL expression may be an independent predictive indicator for GC patients. Furthermore, Sun W et al. held that activated MLKL compromises the integrity of the cancer cell membrane, resulting in the discharge of intracellular pro-inflammatory molecules that could exert anti-tumor effects. Thus, by blocking MLKL-mediated necroptosis, gastric cancer cells might maintain tumor cell growth (Sun et al., 2019). In cancer cells, many critical molecules related with necroptosis are negatively regulated, raising the possibility that cancer cells may be able to resist necroptosis and hence survive. All malignancies, on the other hand, did not show downregulation of necroptosis-related molecules. In glioblastoma patients, increased RIP1 expression hindered p53 induction through activating the NF-κB pathway, and this upregulation was linked with a worse prognosis in this group of patients (Park et al., 2009). Necroptosis can also be involved in the tumor microenvironment to influence cancer progression. Seifert L et al. found in pancreatic ductal adenocarcinoma (PDA) that RIP3 downregulation *in vivo* did not promote tumor progression. Further studies found that RIP3 deletion induced an immunosuppressive tumor microenvironment with reduced infiltration of TAM and its M2-like Arg1+CD206+ subset, and meanwhile found that the lymphocyte infiltration in PDA increased. The microenvironment mediated by RIP3 deletion could suppress tumors (Seifert et al., 2016). Whereas alterations in the expression of these necroptotic molecules might cause changes in human immune surveillance against cancer. It was discovered that RIPK3 regulated NKT cell activity and promoted the generation of antitumor immune responses by these cells. The expression of RIPK1 and the activation of NF-κB were critical for the induction of CD8^+^ T cell adaptive immunity (Newton et al., 2004; Yatim et al., 2015; Gong et al., 2019). This suggested that necroptosis might alter the expression of immune molecules in the microenvironment to affect cancer cell survival.

Changes in necroptosis-related molecules have been found not only in solid tumors but also in hematologic tumors. Höckendorf U et al. found in acute myeloid leukemia mice that cell death induced by RIPK3 and the release of interleukin-1b (IL-1b) which was mediated by inflammasome, limited myeloid leukemogenesis via eliminating transformed cells and promoting differentiation of leukemia-initiating cells (Höckendorf et al., 2016); furthermore in the clinical 125 observed patients in chronic lymphocytic leukemia (CLL) found that the group of patients with low expression of the CYLD, which is a key mediator molecule in the necroptosis process, had a worse prognosis. This showed that CYLD might exert a critical role in the progression of CLL (Wu et al., 2014).

Nonetheless, in DLBCL, it remains to be further discussed whether and how necroptosis is involved in tumorigenesis development. Therefore, in the study, we analyzed DLBCL transcriptomic data to investigate the role of necroptosis in DLBCL. We looked into the mode of necroptosis in DLBCL and its relationship with the prognosis of DLBCL. And we specifically focused on its effect on the immune microenvironment of DLCBL. Our findings may add to our understanding of the role of necroptosis in malignancies and provide new information for the cure of DLBCL.

## Materials and methods

### Data download and processing

The datas in this study were from public GEO databases. We first searched by the search term “Diffuse large B cell lymphoma”, and then by the restrictions 1) Organisms were restricted to “Homo sapiens”; 2) “Expression profiling by array” was the study type that we confined; 3) Sample counts >200; 4) Complete survival information; 5) Treatment with RCHOP regimen, and we finally included three datasets, GSE31312, GSE181063, and GSE10846. In addition, the dataset GSE31312 has the information about the treatment response assessment, where CR means complete remission, PR means partial remission, SD means stable disease, and PD stands for progressive disease.

We utilized the robust multiarray average (RMA) method to normalize the data after downloading the raw CEL files. And we used the “normalizebetweenarrays” function of the “limma” package to remove batch effect. After that, we cleaned the data even further, and the following criteria were employed in the data cleaning process for all three datasets: 1) data without complete survival time and survival status were excluded; 2) to ensure that death was due to tumor as much as possible, we excluded data with overall survival time <30 days; 3) to ensure comparability of patients, we excluded data treated with non-RCHOP regimens. Considering that GSE31312 covered the richest clinical information, we used it as the dataset for our main analysis, and to further perform data cleaning, we excluded sample with missing “IPI score”, “GEP” information in GSE31312. Finally, GSE31312 included 421 patients with DLBCL, GSE181063 included 598 patients, and GSE10846 included 233 patients. Considering batch effects, combining datasets may cause unnecessary bias, so we kept the data independent.

### Identification of DLBCL-associated necroptosis-related clusters

We collected 67 necroptosis-associated genes through “GOBP-NECROPTOTIC-SIGNALING -PATHWAY” and previous studies (Supplementary Material S1) (Zhao et al., 2021). The “GOBP-NECROPTOTIC-SIGNALING-PATHWAY” came from MSigDB (Molecular Signatures Database v7.4). First, we screened necroptosis-related genes with prognostic significance in the GSE31312 dataset by univariate cox regression, and genes with *p* < 0.05 were considered as DLBCL-associated necroptosis-related genes and included in the subsequent analysis. Subsequently, clustering analysis was performed in the GSE31312 dataset using the R package “ConsensusClusterPlus” (Wilkerson and Hayes, 2010) based on the expression of the above included genes. Resampling was performed 1000 times to ensure classification reliability. The clinical correlation study was carried out based on the clusters that had been identified. We used the survivfit function of the R package “survival” to analyze the prognostic differences among the three groups, and used the logrank test method to evaluate the significance of the prognostic differences between different groups of samples. And the proportion of cluster treatment response among different clusters was presented in the stacked histogram. The R package“gglluvial” was used to visualize the relationship between the three clusters and IPI as well as GEP type, and we used Pearson chi square to test the above data.

### Immune infiltration analysis

We first collected “NK cell mediated cytotoxicity” and “T cell receptor signaling pathway” from MSigDB (Molecular Signatures Database v7.4) and scored each patient in GSE31312 using ssGSEA. Subsequently, we analyzed the patients according to necroptosis-related clusters, and compared the differences of the above pathways between the groups, which were presented in the form of box plots.

After that, we tapped into the R package “ESTIMATE” (Yoshihara et al., 2013) to assess the “immune score”, “stromal score” and “tumor purity” of the different clusters. We conducted Kruskal-Wallis test on the three groups and adopted Wilcox test between the two groups to evaluate the significance of the results. What’s more, the differences between individual immune cells in different clusters were evaluated by both the “Cibersort” and “ssGSEA” methods. The B cells and associated cells were removed from the above two analyses in order to rule out relevant influence.

### Identification of differential expression genes in necroptosis-related clusters

The R package “limma” was utilized to find differential genes among different clusters, and genes that met “adj.*p* < 0.001” as well as “log FC = 0” were considered differentially significant for subsequent analysis. After completing the analysis of variance for all combinations, we took the intersection of the obtained results and showed them in the form of a Venn diagram. The similar method has been used to explore related genes in previously published article (Zhang et al., 2020).

We carried out a secondary clustering analysis on the GSE31312 dataset using the R package “ConsensusClusterPlus” based on the intersected differential genes, and resampled 1000 times to ensure classification reliability. Thereafter, we made on PCA analysis on the three clusters to visualize the differences in expression patterns among the three clusters. Similarly, we performed survival analysis on the three clusters to determine differences in patient prognosis between clusters to verify necroptotic functional cluster stability. We then integrated clinical characteristics including Gender, IPI, GEP, and treatment response to demonstrate the differences in gene expression and clinical characteristics among the three clusters in a heat map.

### Construction and validation of a gene prognostic model and the evaluation of prognostic performance

We first applied the univariate Cox regression analysis to screen the cluster-related differential genes with prognostic significance in the GSE31312 dataset with *p* < 0.05 as the standard. Following that, we exploited Lasso-penalized Cox regression analysis to further screen for necroptosis-related genes with the greatest predictive performance and made use of these genes to build a risk score model. Multivariate Cox regression was adopted to further identify independent predictors and calculate regression coefficients. After collecting regression coefficients about every necroptosis-related gene that was significant to prognosis, using the following formula, we derived a risk score for each patient based on the expression of each gene (Carreras et al., 2020):

$$\text{Risk}\;\text{score}\;=\;\left[\left({\text{Exp}}_{\text{gene}1}\;\text{×}\;{\text{coefficient}}_{\text{gene}1}\right)\;+\;\left({\text{Exp}}_{\text{gene}2}\;\text{×}\;{\text{coefficient}}_{\text{gene}2}\right)\;+-+\;\left({\text{Exp}}_{\text{geneN}}\;\text{×}\;{\text{coefficient}}_{\text{geneN}}\right)\right]$$

The best cut-off value was calculated using the “survminer” package, and patients were separated into high and low groups based on this value. The Kaplan-Meier curves (Ranstam and Cook, 2017)were done to compare the OS of patients in both risk categories and the Time-ROC analysis (Kamarudin et al., 2017) was performed to determine the predictive potential of the model. The regression coefficients produced from GSE31312 were then applied to the test dataset, GSE181063 and GSE10846, which included entire clinical information, in order to level the risk scores of patients for external validation.

In view of the clinical characteristics, we integrated the genetic prognostic model grouping with clinical characteristics consisting of gender, IPI, GEP and treatment response, and performed lasso regression, and further selected the prognostic factors by stepwise regression based on the “lambda.min” value, and chose the final model based on the minimum AIC value. The final parameters obtained from the above analysis were used to construct prognostic line plots to forecast the OS of DLBCL patients at 1, 3, 5 and 7 years, and the stability of the model was appraised by time-ROC and calibration curve.

## Results

### Schematic Diagram of the Overall Flow of the study

**FIGURE 1:**
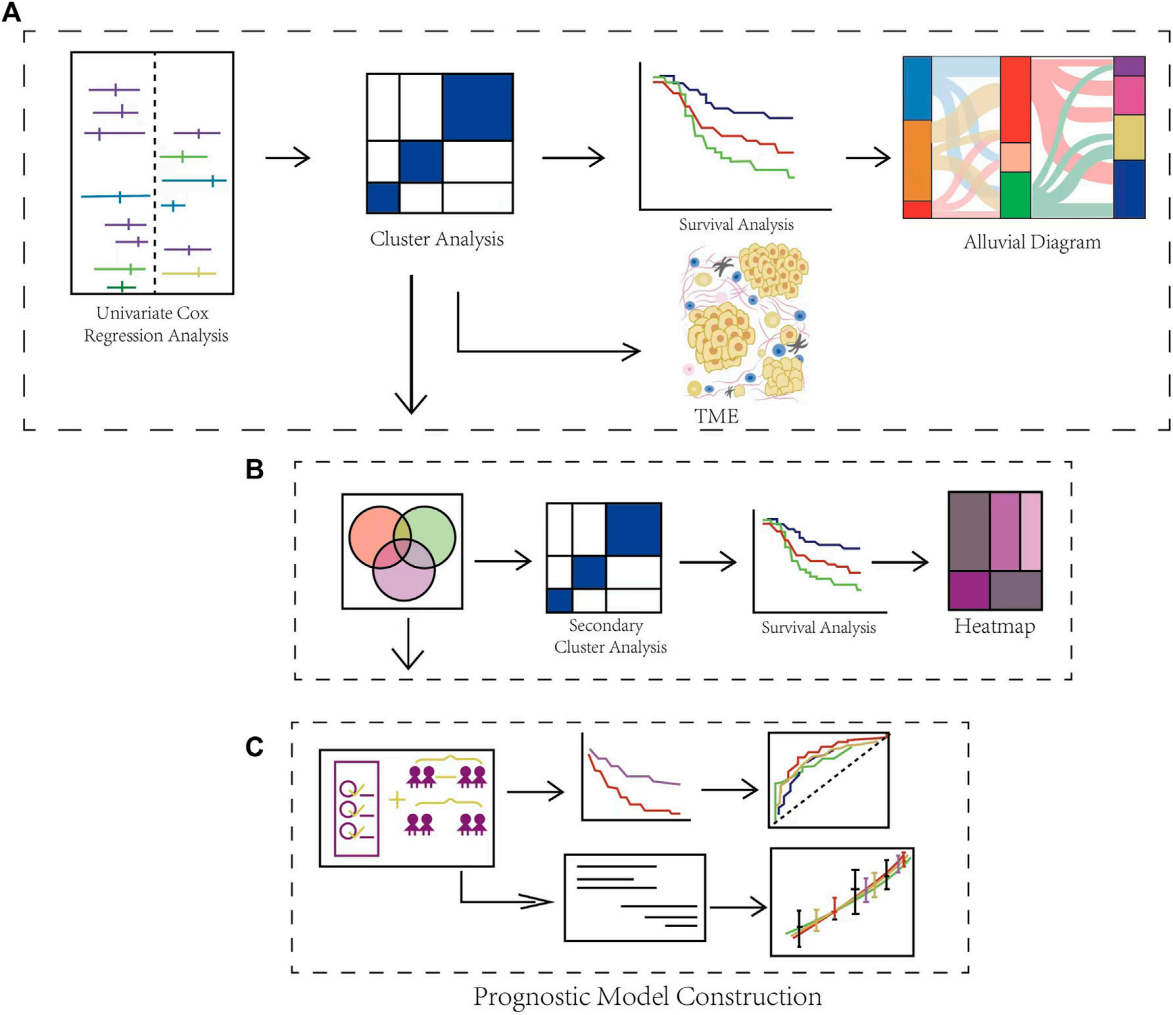
Schematic Diagram of the Overall Flow of the Study. **(A)** Identification of DLBCL-associated necroptosis-related clusters and immune infiltration analysis. **(B)** Identification of differential expression genes in necroptosis-related clusters. **(C)** Construction and validation of a gene prognostic model and the evaluation of prognostic performance.

Figure 1 is the workflow chart of this study, which basically describes the process of this study. First, we screened 17 genes through univariate cox expression analysis in the study. Based on these genes, cluster analysis was carried out to obtain cluster 1, 2 and 3. And then we carried out clinical survival analysis and clinical characteristics analysis on the three clusters. We found that the three groups had heterogeneity in clinical characteristics. So we then analyzed the TME of the three clusters (Figure 1A). In order to screen potentially necroptosis-related genes, we took the intersection of the obtained results through completing the analysis of variance for all combinations in the necroptosis-related clusters. And we performed secondary cluster analysis to obtain cluster A, B and C, and then performed survival analysis on the cluster A, B and C (Figure 1B). Through lasso penalized Cox analysis, 6 prognostic genes were included. Patients were divided into high-risk and low-risk groups according to these genes, and ROC time analysis was carried out. Finally, the clinical prognostic model was constructed, and the predictive nomogram was constructed and verified, and the calibration curve was analyzed (Figure 1C).

### Necroptosis-related clusters identified in DLBCL

**FIGURE 2:**
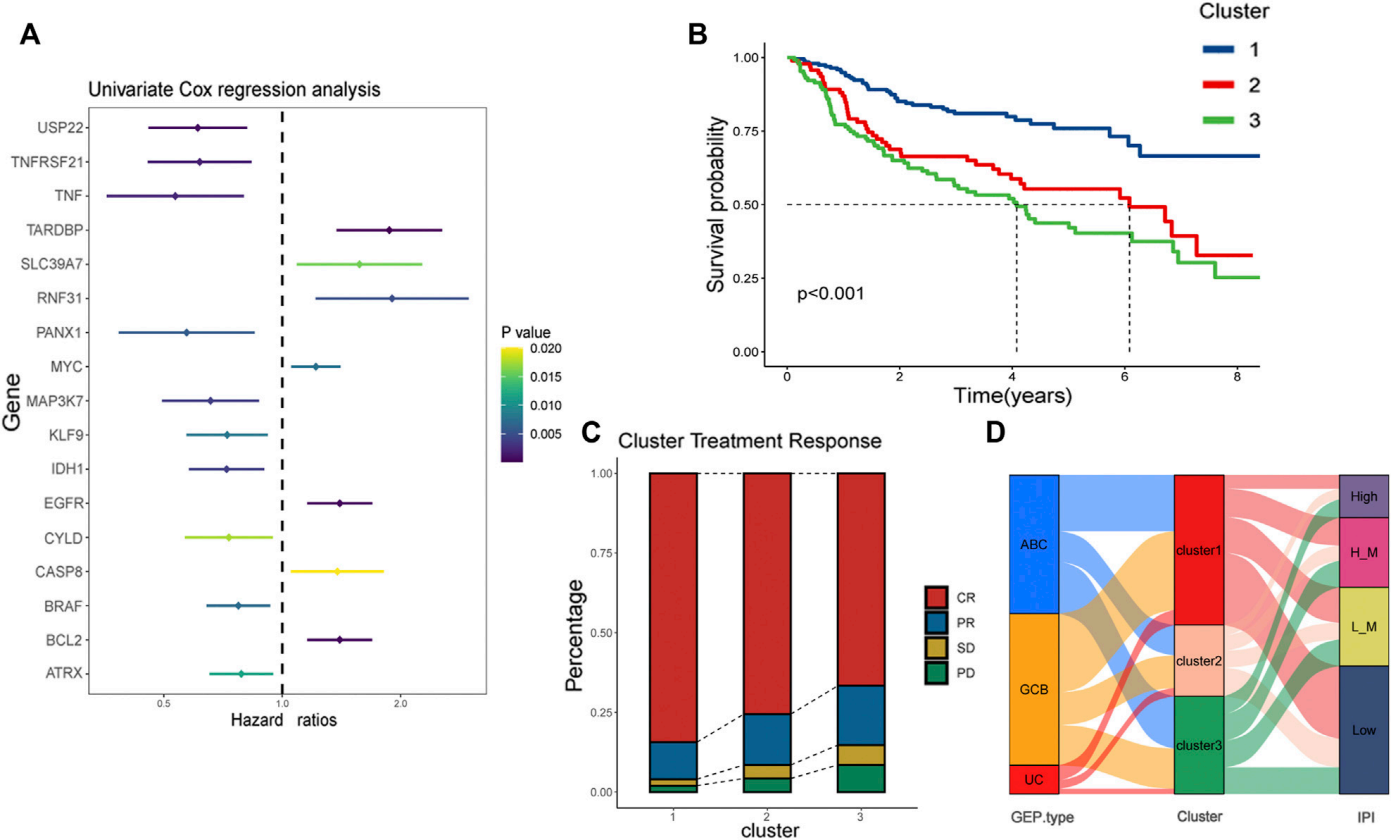
Identification of necroptosis-related clusters and clinical correlation analysis. **(A)** Risk ratios of 17 DLBCL prognosis-related necroptosis genes. Vertical coordinate is gene name, and horizontal coordinate represents risk ratio. Right side is *p*-value range symbolizing that the lighter the color, the larger the *p*-value. **(B)** Kaplan-Meier plots showing the prognosis of three necroptosis patterns in 421 patients from GSE31312. Blue line represents cluster 1, red cluster 2, and green cluster 3. Cluster 1 has the best prognosis. **(C)** Response of patients in the three clusters to RCHOP regimen treatment, with the vertical axis as a percentage. Red represents CR, yellow PR, green SD, and pink PD. **(D)** Alluvial is used to observe the relationship between cluster 1, cluster 2, and cluster 3 with IPI and GEP type. The red part of the middle bar represents cluster 1, pink cluster 2, and green cluster 3. “L_M”means low-intemediate, and “H_M” means high-intemediate.

Firstly, 67 necroptotic genes were collected based on previous studies (Zhao et al., 2021), and necroptotic genes associated with DLBCL prognosis were screened using univariate cox regression analysis, whose resulte showed in the Supplementary Material S1, and genes with *p*-values less than 0.05 were deemed statistically significant. The final 17 DLBCL-related necroptotic genes were screened for the follow-up study. We regarded this group of genes as DLBCL-associated necroptosis-related genes, and the genes with risk ratio less than 1 were USP22, TNFRSF21, TNF, PANX1, MAP3K7, KLF9, IDH1, CYLD, BRAF, and ATRX. We believed that they were potential protective genes. TARDBP, SLC39A7, RNF31, MYC, EGFR, CASP8 and BCL2 were potential risk genes (Figure 2A). According to previous studies, necroptosis had two sides in tumor (McCormick et al., 2016; Strilic et al., 2016), suggesting that necroptosis may also be heterogeneous in DLBCL. In the dataset GSE31312, we used the R package “ConsensusClusterPlus” to cluster 421 patients according to the expression of 17 DLBCL-associated necroptosis-related genes in DLBCL, and for the classification’s reliability, 1000 resamplings were conducted. Finally, three distinctly different clusters, clusters 1, 2 and 3, were determined. Cluster 1 has 198 patients, cluster 2 has 94 patients, and cluster 3 has 129 patients (Supplementary Figure S1A). We further compared the expression of these 17 DLBCL-related necroptotic genes in three clusters. Most potential protective genes were highly expressed in cluster 1, while most potential risk genes were highly expressed in cluster 3 (Supplementary Figures S2D,E). This suggests that cluster 1 may be related to good clinical features and prognosis, while cluster 3 is the opposite. We then compared the prognosis of patients in these three clusters. Cluster 1 had the best prognosis, followed by cluster 2, and cluster 3 had the worst prognosis, indicating that necroptosis may have different modes of action in DLBCL (Figure 2B). Considering that the patients in GSE31312 were all treated with the same RCHOP regimen, the response to the regimen was directly related to the prognosis, so we analyzed the prognosis of the three clusters in which patients’ response to RCHOP regimen treatment was found. The results showed that cluster 1 had the best response to R-CHOP treatment and had the highest CR rate, followed by cluster 2, while patients in cluster 3, who had the poorest prognosis, had the lowest CR rate. In contrast, the rate of patients with progressive disease (PD) after RCHOP treatment reflected an opposite trend, implying that necroptosis may be associated with RCHOP treatment sensitivity, and drug resistance (Figure 2C).

To visualize the relationship between the three clusters and IPI as well as GEP type, we used the package “gglluvial” (Brunson, 2020) to visualize three. And we used Pearson chi square to test the above data. The results showed that the asymptotic significance was less than 0.05, indicating that cluster 1 had the highest proportion of patients with low IPI as well as GCB type among the three clusters (Supplementary Material S4). The results indicated that cluster 1’s possess low IPI scores and GCB type; it has been reported that the prognosis of GCB type is superior to ABC type (Schmitz et al., 2018). This illustrates that cluster 1is associated with favorable survival characteristics (Figure 2D).

### Evaluation of TME

**FIGURE 3:**
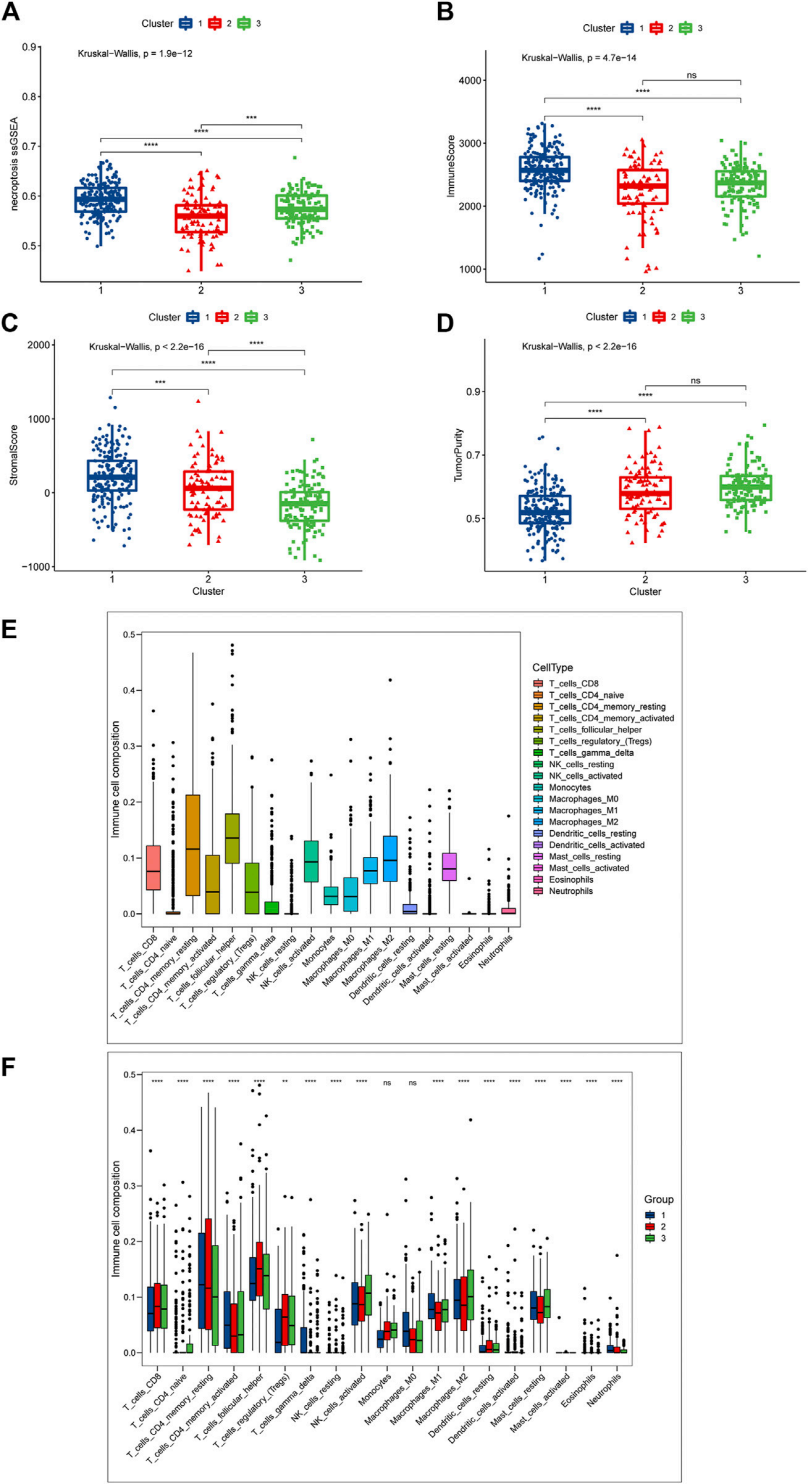
Differences in TME between the three necroptosis-related clusters (Cluster 1, Cluster 2 and Cluster 3). **(A)** Differences in necroptosis scores between the three clusters. **(B)** Differences in immune score. **(C)** Differences in stromal score between the three clusters; **(D)** Differences in tumor purity between the three clusters. **(E,F)** Cibersort was used to assess the infiltration of 19 immune cell types. **(E)** Overall infiltration of 19 immune cells. **(F)** Differences in 19 immune cells between the three clusters. Ns means “not statistically significant”; *p < 0.05; **p < 0.01; ***p < 0.001; ****p < 0.0001 (all significance designations that appear in this paper are minor criteria).

The difference in prognosis suggests that there may be significant heterogeneity between our necroptosis-related clusters, so we first scored the necroptosis of the three clusters through “ssGSEA”, which depicted that cluster 1 had the highest score, cluster 3 the second-highest, and cluster 2 the lowest (Figure 3A). This differs from the prognostic trend, with the best prognosis cluster 1 having the highest level of necroptosis and the worst prognosis cluster 3 scoring higher than cluster 2, explaining that necroptosis might exert a “two-sided” function in DLBCL. The tumor microenvironment (TME) is mainly composed of tumor cells, surrounding immune and inflammatory cells, tumor-related fibroblasts, and nearby interstitial tissues, microtubules, as well as various cell factors and chemokines. It is a complex integrated system, which can be divided into immune microenvironment dominated by immune cells and immune microenvironment dominated by fibroblasts (Fu et al., 2021). Earlier studies have shown that necroptosis is linked to TME, and we speculated that the heterogeneity of necroptosis in DLBCL was correlated with its involvement in influencing the tumor microenvironment. Subsequently, we analyzed the differential profile of several immune-related pathways among the three clusters. The results reflect, for example, that the NK cell mediated cytotoxicity, T cell receptor signaling pathway was significantly activated in cluster 1 (Supplementary Figure S1B). Chances were that the immune microenvironment differed between different necroptosis-related clusters.

To give an insight into the immune microenvironment, we first valued the immune, stromal, and tumor purity between the three clusters by “ESTIMATE”. As shown in Figure 2B, Cluster 1 had the highest immune score, and there was no statistically significant difference between clusters 2 and 3 (Figure 3B); as to the stromal score, cluster 1 had the highest stromal score, followed by cluster 2, and cluster 3 had the lowest stromal score (Figure 3C); cluster 1 had the lowest tumor purity, and there was no significant difference in tumor purity between cluster 2 and cluster 3 (Figure 3D). These data showed that the immunological microenvironment of the three clusters varied significantly, and we then employed the “Cibersort” package to further analyze the differences in immune cell composition among the three clusters. For the purpose of avoiding unneeded interference, we eliminated B cells and associated immune cells from the investigation. We first analyzed the proportion of 19 immune cells in the DLBCL patient population, and found that T cell follicular helper was the highest, followed by T cell CD4 memory resting, and Macrophages-M2 was the third (Figure 3E). Supplementary Figure S1C shows the different proportion of immune cells infiltrated in each patient. Next, we compared the differences in the immune components of the three clusters, and we focused on the aforementioned cells, and the results indicated that compared with other clusters, cluster 1 had the highest relative proportion in T cells CD4 memory resting, T cells CD4 memory activated, and Macrophages-M1, while cluster3 with the worst prognosis had the highest relative proportion in Macrophages-M2. For T cell follicular helper, it was different from the above cells and inconsistent with the trend of prognosis. Cluster 2 was the highest, cluster 3 was the second and cluster 1 was the lowest (Figure 3F).

**FIGURE 4:**
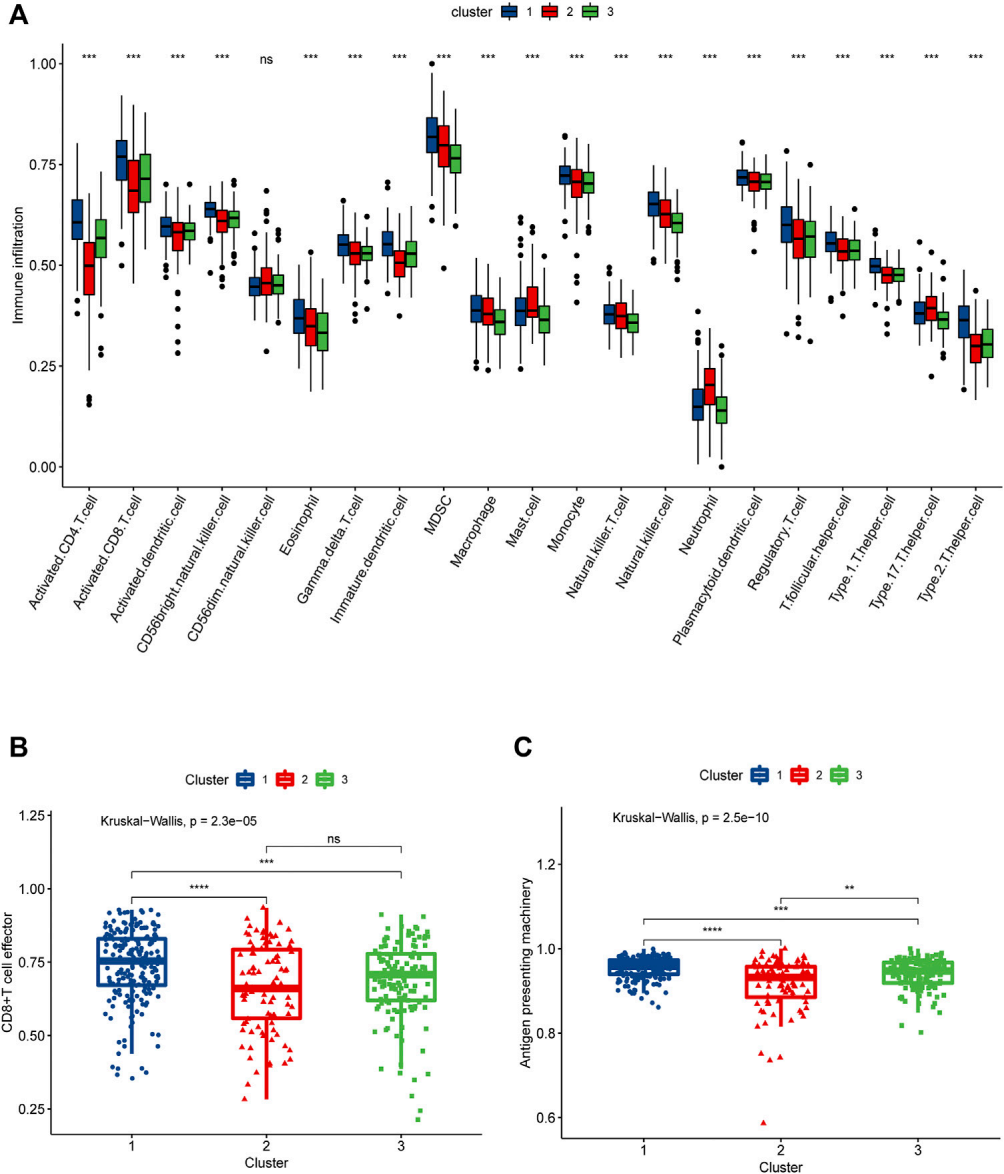
SsGSEA assessment of immune infiltration in three necroptosis-associated clusters (Cluster 1, Cluster 2 and Cluster 3). **(A)** Differences in the abundance of 21 infiltrating immune cells in the three necroptosis-related clusters, with Cluster 1 in blue, Cluster 2 in red and Cluster 3 in green. **(B)** Differences in CD8^+^ T cell effector scores between the three necroptosis-related clusters. **(C)** Differences in antigen presenting machinery scores between three necroptosis-related clusters.

In order to more rigorously evaluate the immune infiltration, we analyzed the abundance of different immune cells infiltration using “ssGSEA”. What we discovered was that cluster1 displayed a high level of adaptive immune activation, such as CD8^+^ T cells as well as CD4^+^ T cells infiltrated abundantly; meanwhile, in the three clusters, the level of infiltration of many immune cells, including T cells CD4 memory activated, NK T cells, and NK cells, was consistent with the prognostic trend, manifesting the highest level of infiltration in cluster 1, followed by cluster 2, and the lowest in cluster 3 (Figure 4A); it was clear that we could notice differences in immune infiltration between the three clusters, and we also evaluated differences in antigen presentation mechanism (APM), which was shown to correlate with T cell infiltration scores (Şenbabaoğlu et al., 2016) and CD8^+^ T cell effector. The results showed that cluster 1 attained a higher activation level than the other two clusters, which verified the high level of immune infiltration in cluster 1 (Figures 3B,C); in addition, we also noted that cluster 2 achieved the highest level of expression in mast cells, cluster1 got the lower level and cluster3 had the lowest. The above findings point towards the idea that necroptosis may have a heterogeneous effect on the immune microenvironment shaping of DLBCL among different functional clusters.

To further explore the correlation between immune cells and genes, we analyzed the correlation between M1, M2,CD8+T cells and 8 classical pathway molecules of necroptosis, as well as 6 modelled genes screened subsequently. The results showed that M1 cells were positively correlated with FAS, MLKL, RIPK1, RIPK3 and ACTB; M2 cells associated with poor prognosis were negatively correlated with SNRPD2,PAICS (Supplementary Figure S2B).

### Identification of differentially expressed genes in necroptosis-related clusters

**FIGURE 5:**
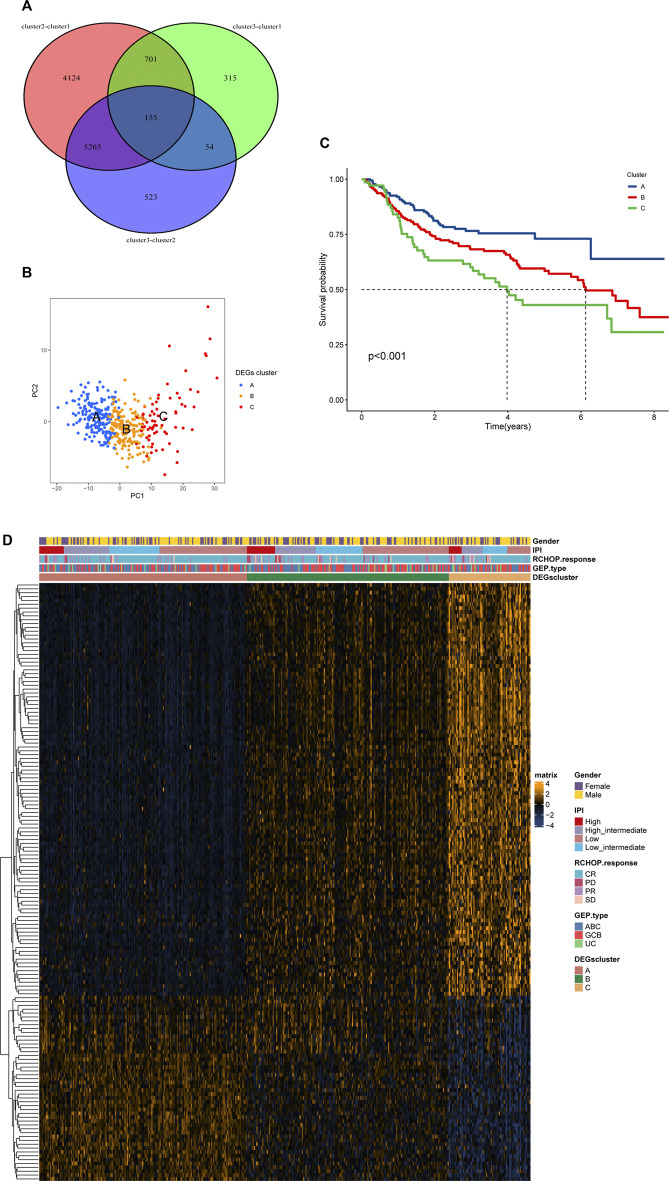
(Continued).

By taking the intersection of differentially expressed genes among the groups, we finally obtained 155 necroptosis-related cluster-associated genes (Figure 5A). We then carried out secondary clustering of GSE31312 based on these 155 genes and again obtained three clusters A, B, C, with 178 people in cluster A, 173 in cluster B, and 70 in cluster C (Supplementary Figure S1D). PCA analysis exhibited significant differences among the three clusters, and subsequent survival analysis observed similar survival differences to those of the necroptosis-related clusters (cluster 1, cluster 2, and cluster 3) — patients in cluster A had the best prognosis of the three, and cluster B had a better prognosis than cluster C (Figures 5B,C). A heat map of 155 genes suggested significantly different expression patterns among patients in the three clusters (Figure 5D). Among them, the expression patterns of Cluster A and Cluster C were almost opposite, and the genes with significantly elevated expression in Cluster A were mostly downregulated in Cluster C. We also analyzed the expression of 8 classical pathway genes of necroptosis among the three clusters, and we found that there was still significant inter-cluster heterogeneity. Most of the genes were expressed highest in cluster A and lowest in cluster C. It could be seen in Supplementary Figure S1E that TLR3, in contrast to the prognostic trend, experienced a gradual increase in expression level in the three clusters of A, B and C. Hence, we think that there may be stable differences in the mode of action of necroptosis in DLBCL.

### Prognostic model construction of necroptosis-related genes

**FIGURE 6:**
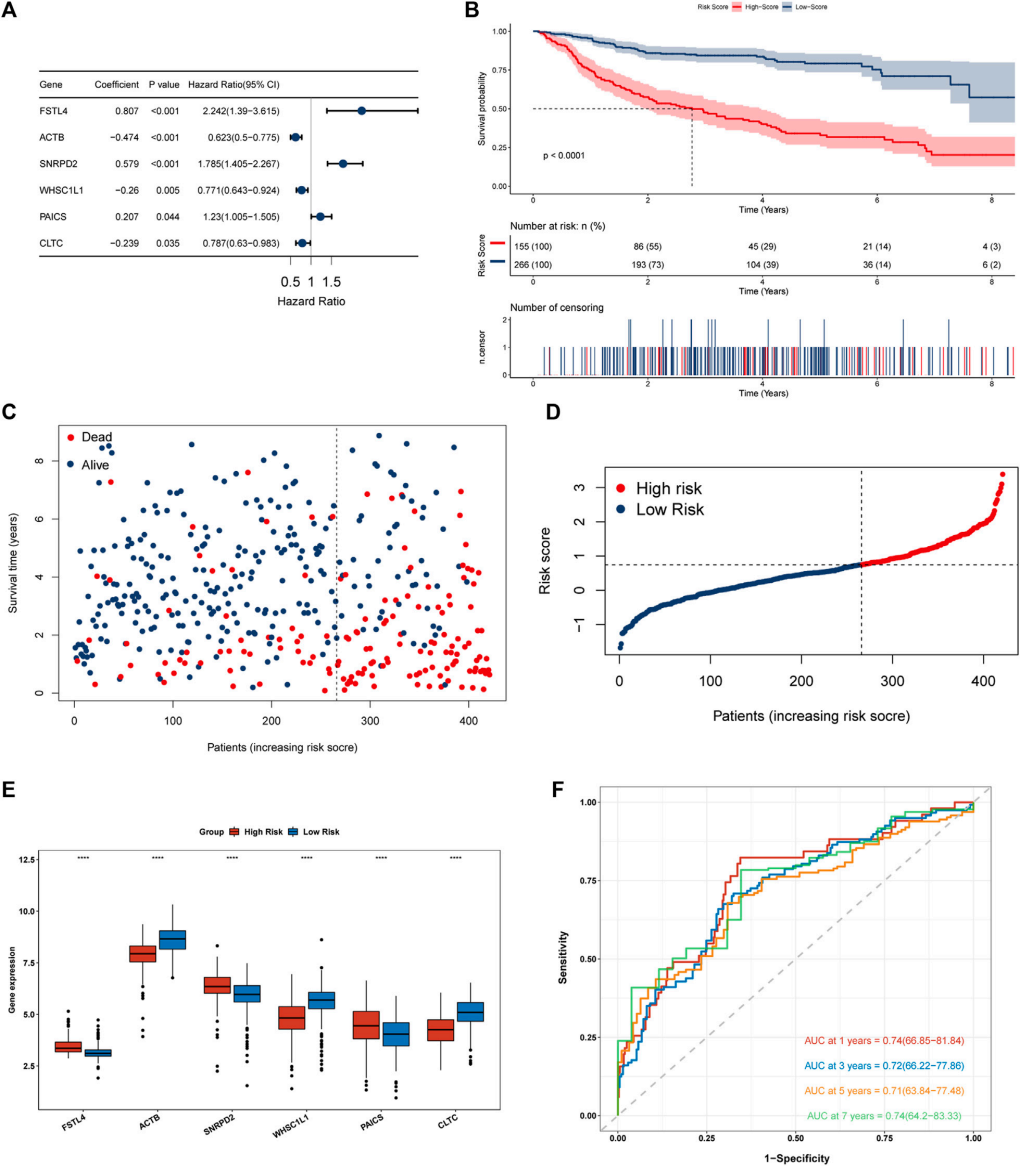
Construction and evaluation of a 6 necroptosis-related genes prognostic model. **(A)** Forest plot of 6 genes multivariate cox regression. **(B)** Kaplan-Meier survival analysis for the training set (GSE31312). **(C,D)** Heat map of risk scores and survival status of 421 DLBCL patients in the training dataset. **(E)** Differential expression of 6 modeled genes in the high and low risk groups in the training dataset, where red box represents high risk and blue represents low risk. **(F)** A Time-ROC curve analysis of the signature in training dataset.

**FIGURE 7:**
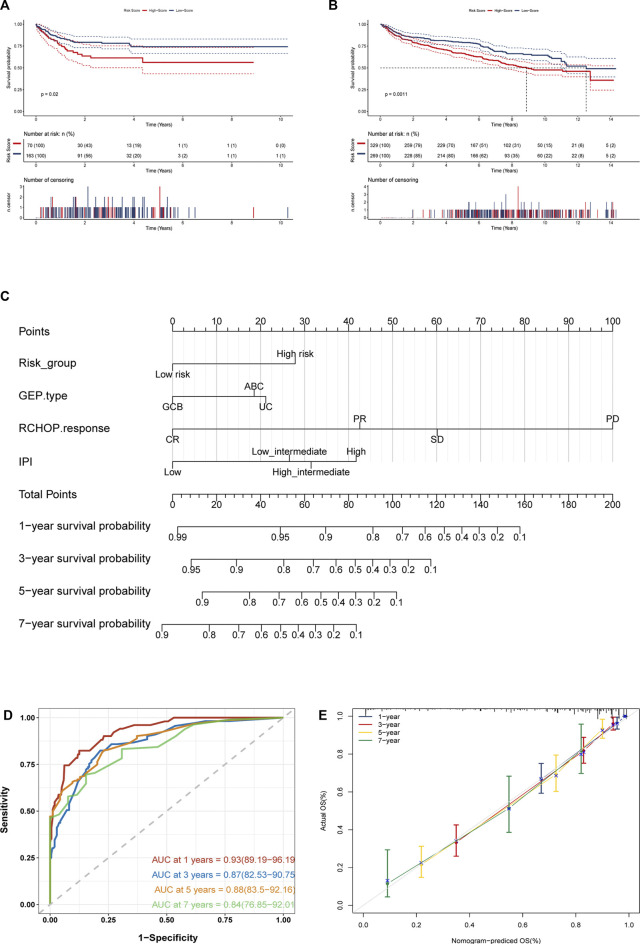
(Continued).

The study included 421 patients from the training dataset GSE31312.74 prognosis-related cluster differential genes were first screened by univariate cox regression and then further selected by Lasso-penalized Cox analysis. Eventually, 6 prognosis genes were included and their regression coefficients—FSTL4, ACTB, SNRPD2, WHSC1L1, PAICS, and CLTC—were calculated. From the forest plot, FSTL4, SNRPD2 and PAICS could be potential oncogenes, while ACTB, WHSC1L1, and CLTC were thought as protective genes (Figure 6A). Next, each patient’s risk score was computed using the following formula:

[(Exp FSTL4 × (0.807) + (Exp ACTB × (−0.474) + Exp SNRPD2 × (0.579) + Exp WHSC1L1 × (−0.26) + Exp PAICS × (0.207) + Exp CLTC × (−0.239)]. GSE31312 patients were classified into two groups according to their best cut-off values. The low-risk group had much superior OS than the high-risk group, as seen by Kaplan-Meier survival curves. (Figure 6B). The risk score was significantly connected with prognosis. As the score increased, the mortality rate of patients jumped (Figures 6C,D). We then evaluated the differential expression of 6 genes in the two risk groups and found that the expression of FSTL4, SNRPD2 and PAICS grew in the high-risk group, while the expression of ACTB, WHSC1L1 and CLTC went up in the low-risk group, which further confirmed the reliability of the selected prognostic genes (Figure 6E). In order to further verify the reliability of the genes we screened, we evaluated the differential expression of the 6 genes in clusters 1, 2 and 3. The results showed that SNRPD2 and PAICS, which represents poor prognosis in, creased in cluster3, while fstl4 had the lowest expression in cluster1 group with good prognosis. Besides, the expression of ACTB, WHSC1L1, and CLTC, which represent good prognosis, increased in cluster 1, further confirming the reliability of the screened prognostic genes (Supplementary Figure S2A). We then integrated the expression of necroptosis-related genes, the expression of 6 prognostic genes, and risk score data to construct a correlation matrix, as shown in Supplementary Figure S1F, 6 prognostic genes were firmly linked with the classical pathway molecules of necroptosis. Besides, ACTB, and FSTL4 showed a completely opposite relationship. FSTL4 was positively linked to FAD, FASLG, and TLR3, and negatively connected with FAS, MLKL, RIPK1, and RIPK3, while ACTB had a positive correlation with FAS, MLKL, RIPK1, and RIPK3, and inverse relation to TLR3 and FASLG. Consequently, we have reason to conjecture that ACTB and FSTL4 are mutually antagonistic necroptosis-related genes (Supplementary Figure S1F). The time-ROC curves depicted that the AUC of OS predicted by the genetic prognostic model was 0.74, 0.72, 0.71, and 0.74 at 1, 3, 5 and 7 years respectively, which were all greater than 0.7, demonstrating the good prognostic ability of the model (Figure 6F).As presented in the Kaplan Meier curves in the two external validation sets GSE10846 and GSE181063, compared to the low-risk group, the prognosis for the high-risk group was much poorer. (Figures 7A,B), suggesting that the model was stable.

### Construction and validation of a predicted nomogram

Considering the excellent predictive prognostic ability of gene models, we further explored their role in clinical applications. We included several clinical features that were routinely considered prognostically significant in clinical practice and were available. Based on the results of lasso combined with stepwise analysis, we finally incorporated necroptosis-related genetic model risk groups, GEP type, RCHOP treatment response, and IPI scoring to construct a prognostic nomogram (Figure 7C). The C-index of the nomogram was 0.840. And the Time-ROC analysis showed that its AUCs for predicting OS at 1, 3, 5, and 7 years are 0.93, 0.87, 0.88, and 0.84, respectively (Figure 7D). According to a further inspection of the calibration curve, the predicted OS was generally consistent with the observed OS (Figure 7E). In conclusion, the above results indicated that the nomogram was successful in forecasting DLBCL patients’ survival time.

## Discussion

DLBCL, the most prevalent kind of NHL, is a heterogeneous group of diseases with varying biologic characteristics, clinical manifestations, and therapeutic responses. While 2/3 of DLBCL patients respond well to R-chop regimens, there is still a lack of effective treatment for patients with secondary or partial primary drug resistance. Therefore, it is essential to find new research directions for DLBCL patients (Coiffier et al., 2010). Necroptosis, as a combination of apoptosis and necrosis (Tang et al., 2020), is a programmed cell death mediated mainly by RIPK1, RIPK3, and MLKL (Pasparakis and Vandenabeele, 2015; Gong et al., 2019). Currently, necroptosis has been found to be closely associated with tumors, such as colorectal cancer (Feng et al., 2015; Bozec et al., 2016), gastric cancer (Ertao et al., 2016; Sun et al., 2019), glioblastoma (Park et al., 2009), and pancreatic ductal adenocarcinoma (Seifert et al., 2016), whose involvement in the tumor microenvironment to influence the development of cancer has been demonstrated (Seifert et al., 2016). In addition, necroptosis, reportedly, played a role in the development of hematologic neoplasms. RIPK3 downregulation could accelerate leukemogenesis in acute myeloid leukemia mice, and afterward they found that RIPK3-mediated cell death could curtail the production of myeloid leukemia *via* eliminating transformed progenitor cells and promoting differentiation of leukemia-initiating cells (Höckendorf et al., 2016); in a clinical cohort study, chronic lymphocytic leukemia patients with low expression of the CYLD gene were observed to have a worse prognosis; before that, CYLD was noticed to interfere with several key tumor-associated signaling pathways, particularly the NF-κB pathway, by regulating the ubiquitination status of its components, and thus the low expression of CYLD impaired the ability to inhibit CLL tumor progression leading to a worse prognosis (Kovalenko et al., 2003; Wu et al., 2014). Yet, in DLBCL, whether and how necrotic apoptosis is involved in tumor development remains to be investigated, so our work was aimed to elucidate the function of necroptosis in DLBCL by analyzing DLBCL transcriptomic data.

In our research, three different necroptosis-related clusters were identified according to 17 necroptosis-related genes. We noted significant prognostic differences between the different clusters. The best prognosis was found in patients in cluster 1, while the poorest prognosis was seen in those in cluster 3. This reflected that heterogeneity may exist among the three clusters. Furthermore, cluster 1 was shown to have a GCB phenotype and a low IPI score compared to the other two clusters, which explained that cluster 1 may display favorable clinical characteristics. Following that, we re-obtained new three clusters, Cluster A, Cluster B, and Cluster C, based on the secondary clustering analysis of necroptosis-related differential genes in the three clusters mentioned above. The survival analysis results were similar to the first cluster analysis, and the expression of classical molecules of necroptosis in the three clusters was significantly heterogeneous, indicating that necroptosis may indeed manifest itself in distinct patterns in DLBCL.

RCHOP is the primary therapy for DLBCL, and a patient’s reaction to medications is directly tied to their prognosis (Tilly et al., 2015). Our result of medication response is compatible with the distinct prognostic aspects of each cluster; whereas the rate of patients with PD showed an inverse tendency, suggesting that necroptosis may mediate RCHOP treatment sensitivity and resistance; in addition high necroptosis scores had lower mortality in cluster 1 than in cluster 2, yet cluster 3 had a higher mortality rate than cluster 2, which had the lowest necroptosis score. This demonstrated that necroptosis assumed a “double-edged sword” role in DLBCL.

The role of necroptosis in TME is increasingly recognized, as the immune microenvironment it created was linked to the development, metastasis, immunity and differentiation of many tumors (Tang et al., 2020). Therefore, by analyzing the immune infiltration of the three clusters, we noticed an abundant immune microenvironment component and a lower percentage of tumor cells in cluster 1while cluster 3 was opposite. Moreover, we observed that cluster 1 had the highest abundance of NK T cells, dendritic cells, and CD8^+^ T cells among the three clusters by analyzing the immune components, and we noted that cluster 1 had a high relative proportion of Macrophages M1. Previously, necroptosis was found to promote the immunity of NKT cells by increasing RIP3 gene expression and activating PGAM5, which exerted a tumor suppressive function (Kang et al., 2015). After that, Paul S et al. also found that activation of NKT cells regulated the frequency of M1 macrophages and Th1 cells effector in secondary lymphoid tissues, further stunting tumor growth (Paul et al., 2019); reduced antigen presentation turned out a mechanism of tumor immune escape including inhibition of dendritic cell antigens, interference with antigen processing and presentation, but necroptosis, by the release of DAMPs, could activate dendritic cell releasing cytokines that activate adaptive immune to suppress tumors (Pasparakis and Vandenabeele, 2015; Jhunjhunwala et al., 2021). In addition, Rosenberg et al. found through exploratory translational analysis that the gene expression of CD8 T cell effector was linked with PD-L1 immunohistochemical expression on tumor-infiltrating immune cells (Rosenberg et al., 2016). Thus we considered that necroptosis in cluster 1 acted as a tumor suppressor in DLBCL relying on inducing enhanced activation and infiltration of the immune component for improved prognosis, and analysis of APM and CD8^+^ T cell effector indicated that cluster 1 may be responsive to anti-PD-1/PDL-1 therapy. We also noted in particular that in cluster 1 high levels of NK cell infiltration, and numerous experiments demonstrated that NK cells functioned effectively in fighting transformed and malignant cells (Hodgins et al., 2019), and in a clinical study by low NK cell counts could contributed to impaired R-CHOP response and increased risk of cancer recurrence Zare et al. (2020). In our analysis, patients in cluster 1 had the best therapeutic response to RCHOP, while patients in the other two clusters with less NK cell infiltration than cluster 1 had a worse therapeutic response. Therefore, it was reasonable to guess that necroptosis-induced high NK cell infiltration might potentially impact the responsiveness of R-chop treatment in individuals with DLBCL.

M2 macrophage was found to promote tumor cell survival, invasion, metastasis and angiogenesis, and their increased numbers gave rise to poor prognosis in patients (Pollard, 2004; Najafi et al., 2019). Some investigators found in patients with rectal cancer a potential link to increased tumor resistance to anticancer drugs by M2 (Lan et al., 2021). Such a prognostic relationship was seen in malignant lymphoma as well. Nam SJ, et al. found in a retrospective study of patients with follicular lymphoma that Tumor-associated macrophage (TAM) was observed at higher levels in the poor prognosis group than in the good prognosis group, suggesting that the downregulation of genes associated with macrophage activity in the mRNA transcriptome predicted a favorable outcome. What’s more, such TAM had a phenotype and function that was similar to that of M2 macrophage (Nam et al., 2016). The team subsequently identified M2 in DLBCL as a possible significant predictor of poor patient prognosis (Nam et al., 2018). In our study, we found that the cluster 3 with the worst prognosis possessed the highest M2 infiltration, on the one hand further confirming that M2 took a negative role in the microenvironment of DLBCL, and on the other hand, notably, necroptosis in the cluster 3 characterized by relatively high levels of necroptosis seemed to play a role in contrast to cluster 1 which induced inhibition of the tumor microenvironment. Targeting TAM therapy including repolarization of TAM from M2 to M1 phenotype was gaining attention (Zheng et al., 2017) and in our study we found different levels of M1/M2 cell infiltration in different necroptotic functional clusters, and targeting necroptosis to induce M2 to M1 could be a promising therapeutic idea.

Meanwhile, we found the lowest abundance of mast cell infiltration in cluster 3 and the highest in cluster 2. Mast cells were reported to suppress immunity and promote tumor growth by releasing pro-angiogenic cytokines, interleukins and other cells in DLBCL patients, but previous studies showed that mast cell infiltration was a favorable prognostic factor in DLBCL (Hedström et al., 2007; Marinaccio et al., 2016). We believed that there was a certain association between mast cells and the reason why high levels of necroptosis in cluster 3 did not bring better survival than cluster 2. In other words, appropriate levels of mast cells could exert a positive effect in the TME microenvironment of DLBCL patients towards a good prognosis, while too high or too low levels of mast cells may play the opposite role. Notwithstanding this role in TME of DLBCL was not clear, it would a direction of our future research that deserved attention.

We also found that necroptosis-related genes were dependable predictors of prognosis. Our study identified six potential necroptosis genes of prognostic value, among which the ACTB-FSTL4 antagonistic relationship might be related to the mode of action of necroptosis in DLBCL, for patients with high expression of ACTB possessed a better prognosis, while upregulation of FSTL4 tended to indicate a poor prognosis. Previously, ACTB, in head and neck squamous carcinoma (HNSCC) and other cancers, was found to impact tumor metastasis as well as tumor invasion through NF-κB and Wnt-β-catenin protein pathways (Frontelo et al., 1998; Rubie et al., 2005; Popow et al., 2006); Wright A et al. reported that CYLD could limit the persistent activation of NF-κB signaling by deubiquitinating RIPK1, thus activating necroptosis-related pathways (Wright et al., 2007; Gong et al., 2019). Nonetheless, whether ACTB is engaged in the activation or inhibition of the necroptosis pathway in DLBCL by influencing RIPK1-mediated changes in the NF-κB pathway has not yet been investigated, and this is a direction worth exploring. Meanwhile, the 6 necroptosis-related gene prognostic model could successfully differentiate between high- and low-risk individuals of DLBCL. Such differences were validated in an external independent validation set, proving that the model was reliable. Further integration of clinical features could further appreciate the prognostic significance of the model, and the predictive prognostic nomogram constructed after combining with prognosis-related clinical features could effectively achieve individualized risk assessment.

However, our study has shortcomings. First, our study is based on a public database and lacks further validation of an independent prognostic cohort, and we included patients with baseline data prior to RCHOP treatment, and whether necroptosis is involved in tumor killing by the RCHOP regimen remains to be further investigated. Furthermore, the role of necroptosis in DLBCL still needs to be validated by further experiments.

## Conclusion

In summary, our study found different modes of action of necroptosis in DLCBL, especially in the impact on TME. Clusters that induced abundant immune cell infiltration had a better prognosis, whereas clusters with a poorer immune microenvironment component had a worse prognosis. Increased necroptosis-induced NK cell infiltration promised a better response to RCHOP treatment, while increased induction of M2 cell infiltration indicated a potential poor prognostic factor. The position of necroptosis in DLBCL could not be ignored, and a proper understanding of its role remained a worthwhile direction for our future studies. Systematic assessment of necroptosis patterns in DLBCL patients will facilitate our understanding of the cellular infiltration characteristics of the tumor microenvironment and the establishment of personalized therapy for DLBCL patients.

## Data Availability

The original contributions presented in the study are included in the article/Supplementary Material, further inquiries can be directed to the corresponding authors.
