# Supplementary figures and images for: Integrative analysis of a necroptosis-related gene signature of clinical value and heterogeneity in diffuse large B cell lymphoma
Source: Front Genet. 2022 Aug 11;13:911443. doi: 10.3389/fgene.2022.911443 (PMC9403718; doi:10.3389/fgene.2022.911443)

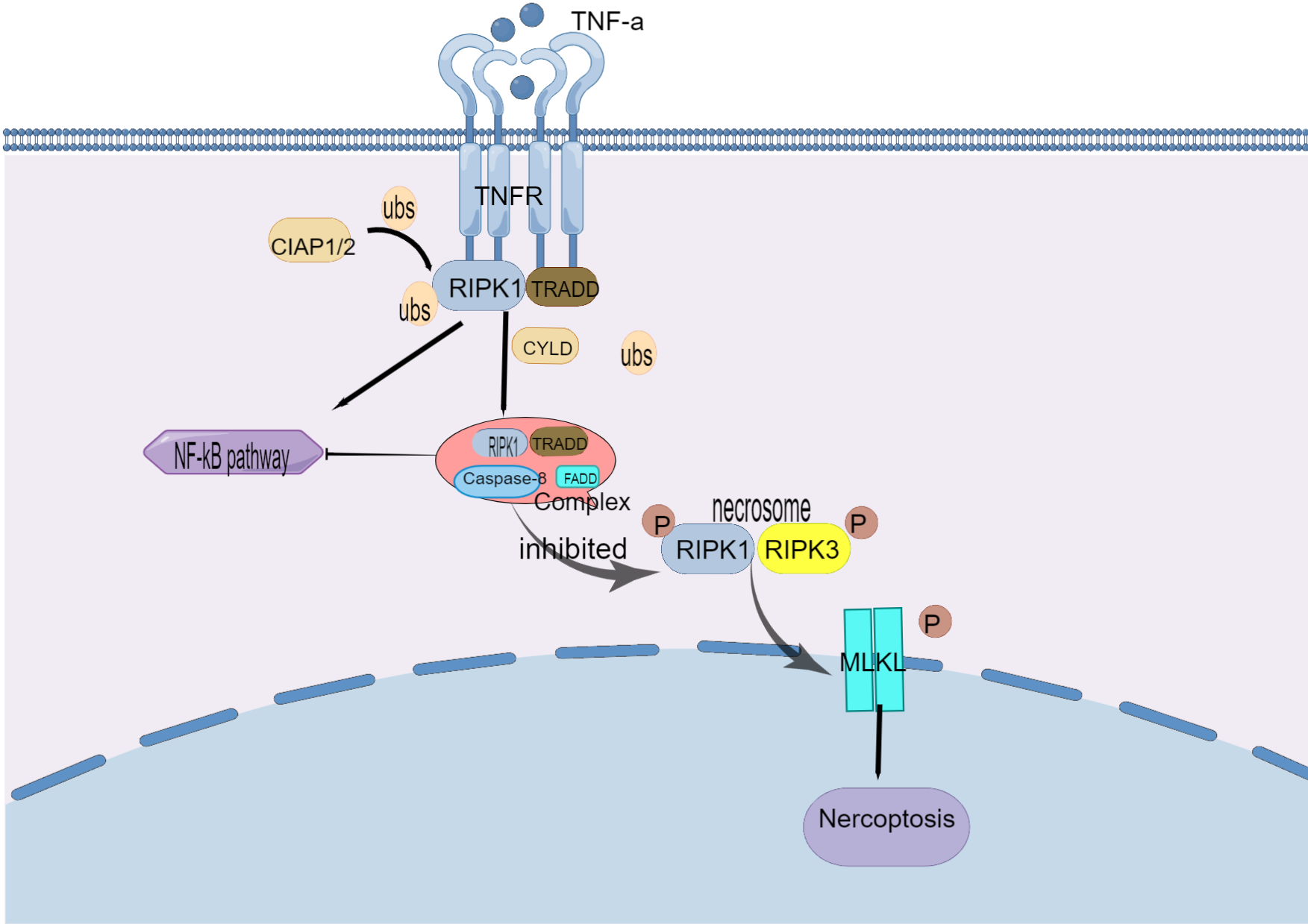

Supplement: Supplementary file 1 [file Image3.TIFF]

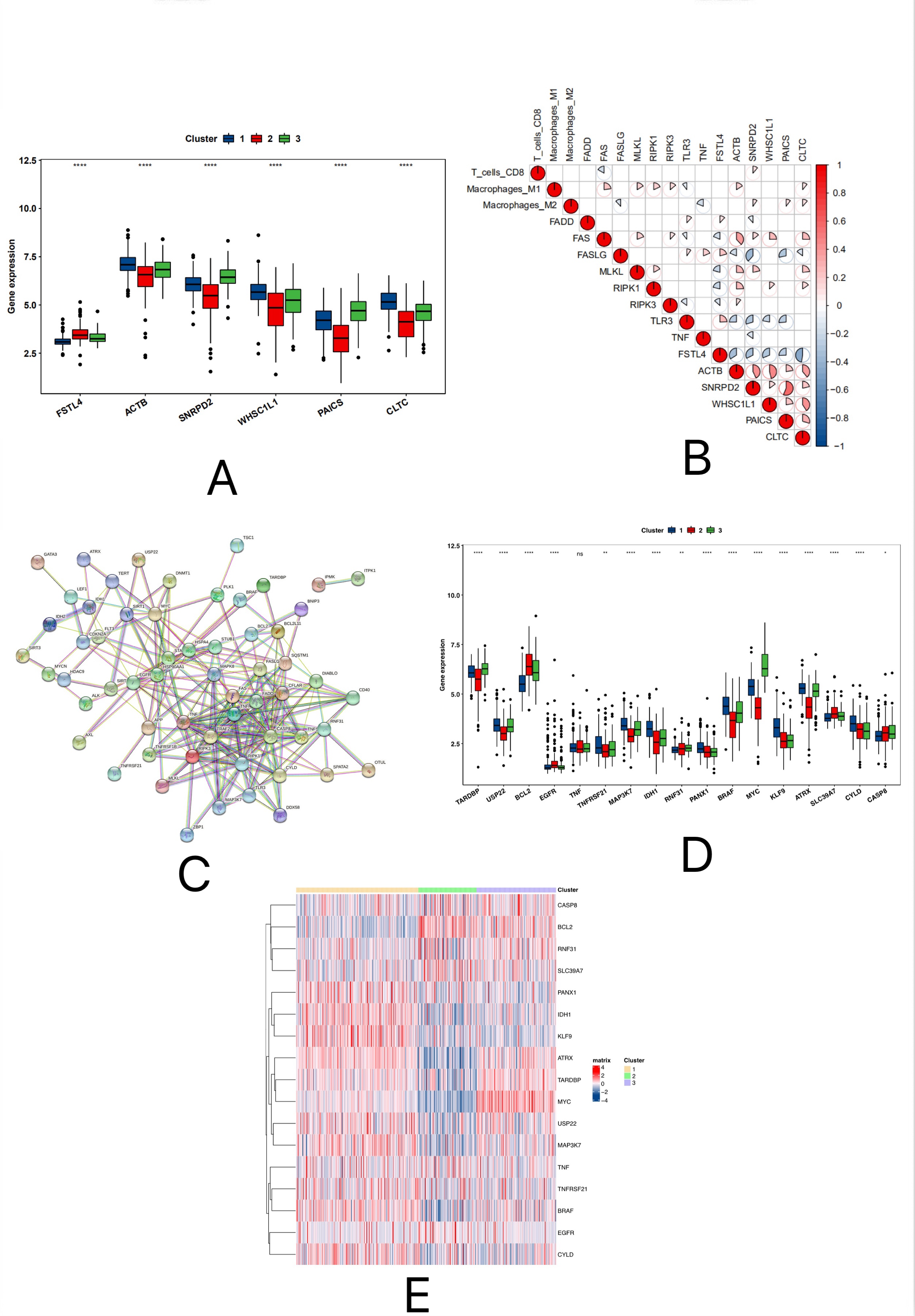

Supplement: Supplementary file 3 [file Image2.TIF]

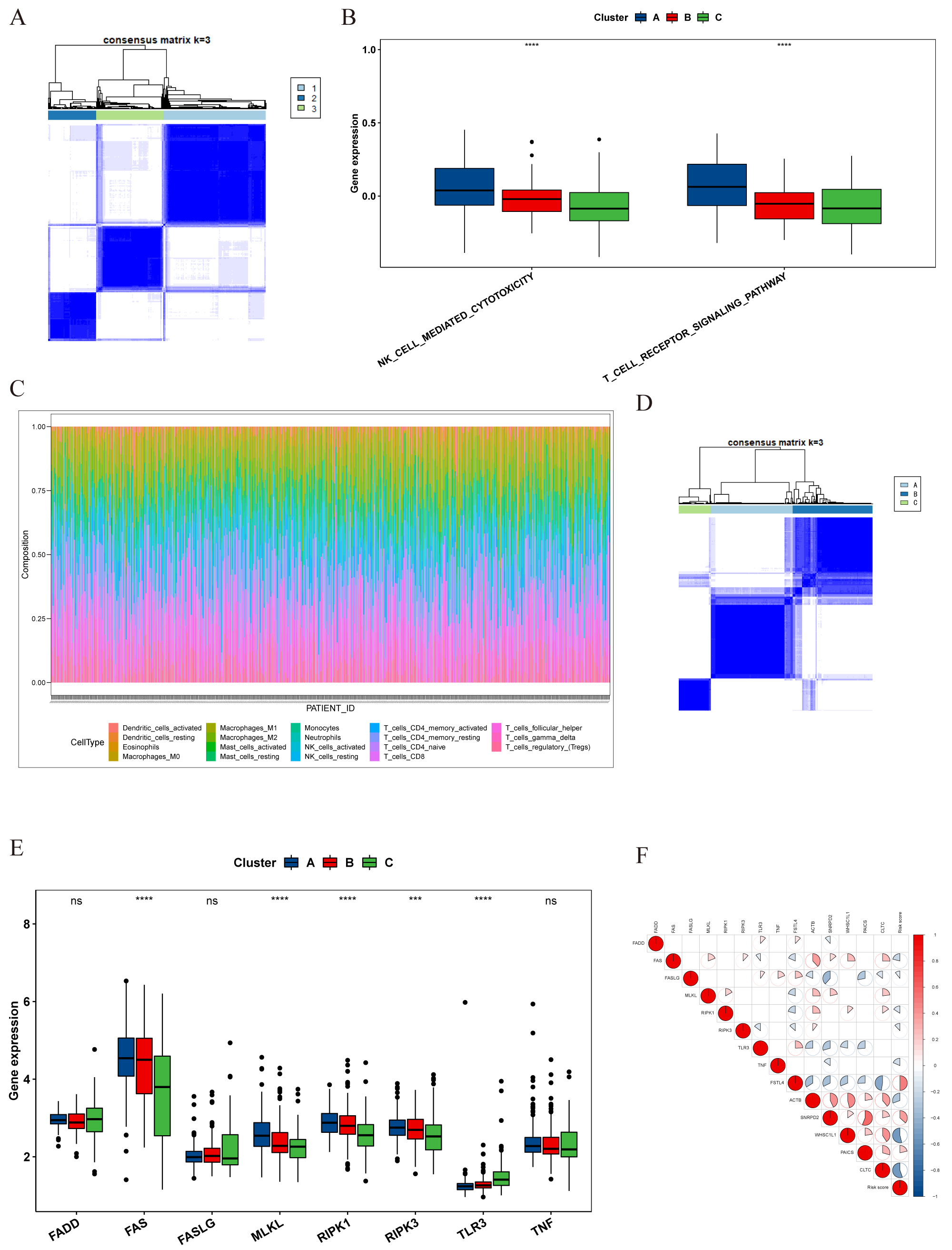

Supplement: Supplementary file 4 [file Image1.TIF]
