# Supplementary material for: Integrative analysis of a necroptosis-related gene signature of clinical value and heterogeneity in diffuse large B cell lymphoma
Source: Front Genet. 2022 Aug 11;13:911443. doi: 10.3389/fgene.2022.911443 (PMC9403718; doi:10.3389/fgene.2022.911443)
Supplement: Supplementary file 7 [file Table2.DOC]

| **Tumor** | | **Association with necroptosis** | **Report** |
| --- | --- | --- | --- |
| **solid tumor** | colorectal cancer | RIPK3 | [9,10] |
|  | gastric cancer | MLKL | [11,12] |
|  | Glioblastoma | RIPK1 | [13] |
|  | Pancreatic ductal adenocarcinoma (PDA) | RIPK3 | [14] |
|  | | | |
| **Hematopathy** | acute myeloid leukemia | RIPK3 | [18] |
|  | chronic lymphocytic leukemia | CYLD | [31] |
